# Dihydroartemisinin decreases pre-existing neutralizing antibodies against adeno-associated virus in challenged mice

**DOI:** 10.3389/fphar.2025.1587135

**Published:** 2025-08-01

**Authors:** Jingjing Fang, Enze Cui, Jinyan Xie, Xuxia Gao, Yun He, Ming Yang, Sana Shaheen, Zhengjun Zhou, Shaolai Zhou, Binbin Cheng, Changquan Ling, Chen Ling

**Affiliations:** ^1^ Oncology Department of Traditional Chinese Medicine, Changhai Hospital, Naval Medical University, Shanghai, China; ^2^ State Key Laboratory of Genetics and Development of Complex Phenotypes and Engineering Research Center of Gene Technology (Ministry of Education), School of Life Sciences, Zhongshan Hospital, Fudan University, Shanghai, China; ^3^ Department of Liver Surgery and Transplantation, Liver Cancer Institute, Zhongshan Hospital, Fudan University, Shanghai, China; ^4^ Faculty of Traditional Chinese Medicine, Naval Medical University, Shanghai, China; ^5^ Shanghai Key Laboratory of Gene Editing and Cell Therapy for Rare Diseases, Fudan University, Shanghai, China; ^6^ Institutes of Integrative Medicine, Fudan University, Shanghai, China

**Keywords:** gene therapy, adeno-associated virus vector, neutralizing antibody, immunosuppression agents, traditional Chinese medicine, dihydroartemisinin

## Abstract

**Introduction:**

The high prevalence of pre-existing neutralizing antibodies (NAbs) against adeno-associated virus (AAV) poses a major obstacle to in vivo gene therapy. Current immunosuppressive (IS) strategies, such as corticosteroids, are limited by toxicity and adverse effects. To explore safer alternatives, we evaluated dihydroartemisinin (DHA), a synthetic derivative of artemisinin inspired by traditional Chinese medicine (TCM), as a potential IS agent.

**Methods:**

In vivo experiments were conducted by administering DHA at either 30 or 210 days post-injection (PI) of rAAVDJ vectors. Anti-AAV NAb levels, transgene expression, and vector genome biodistribution were assessed. Flow cytometry was used to quantify CD20^+^ B cells, germinal center B cells, and plasma cells in the spleen. Splenic gene expression profiling, liver histology, and serum biochemical analyses were performed to evaluate immunological and safety responses. In vitro, the impact of DHA and its serum metabolites on rAAV infection efficiency was tested in HEK293 cells.

**Results:**

DHA administration significantly reduced anti-AAV NAb levels without compromising transgene expression or vector genome distribution. DHA treatment resulted in a reduction of splenic CD20^+^ B cells, germinal center B cells, and plasma cells, alongside changes in splenic gene expression profiles. Liver histology and serum markers confirmed that DHA at 125 mg/kg/day did not induce hepatotoxicity. In vitro assays demonstrated that DHA and its blood metabolites did not interfere with rAAV infection of HEK293 cells across multiple serotypes.

**Discussion:**

These findings suggest that DHA is a safe and effective agent for modulating humoral immune responses to AAV vectors. Our results provide proof-of-concept evidence supporting the use of TCM-derived compounds to address immunological barriers in gene therapy.

## Graphical Abstract

**Figure:**
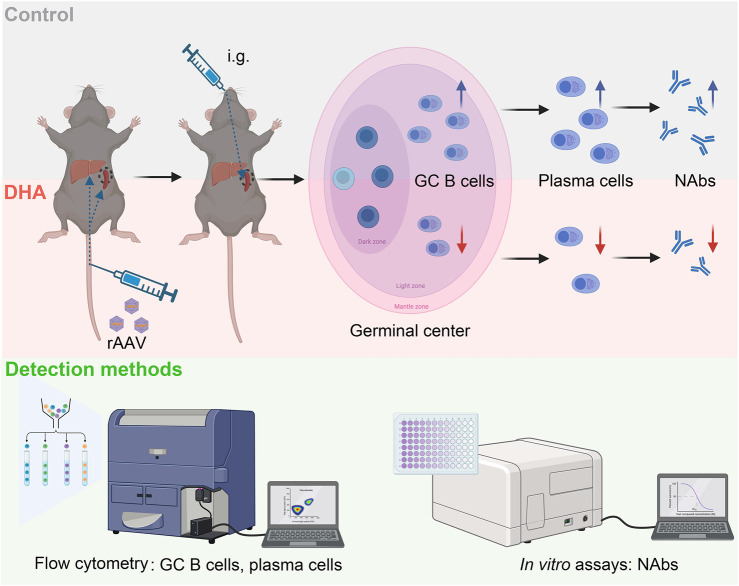
We investigated whether DHA could reduce pre-existing AAV neutralizing antibodies by modulating the humoral immune response. DHA treatment led to a reduction in splenic GC B cells and plasma cells, which are key contributors to antibody production. This immunosuppressive effect was associated with decreased anti-AAV NAb levels, suggesting that DHA could expand the applicability of rAAV gene therapy to a wider patient population and potentially facilitate the re-administration of rAAV-based treatments.

## Introduction

Recombinant adeno-associated virus (rAAV) has emerged as a preferred vector for gene therapy due to its safety, effectiveness, and low immunogenicity, as well as its broad range of serotypes that enable tissue-specific gene delivery (Yang et al., 2024; Sai et al., 2024). The clinical applications of rAAV vectors encompass a variety of genetic disorders, with several rAAV-based gene therapies already approved by regulatory agencies (Mendell et al., 2017). However, despite significant advancements, a major challenge in the successful deployment of rAAV vectors is the presence of neutralizing antibodies (NAb) in patients’ serum, which can severely limit the efficacy of gene therapy (Li et al., 2023).

NAb are generated following natural infection or exposure to rAAV-based treatments. These antibodies target specific epitopes on the capsid of rAAV vector, neutralizing it upon administration, preventing it from reaching target tissues, and inhibiting its ability to transduce cells and to deliver the therapeutic gene. A significant portion of the population has pre-existing NAb against various rAAV serotypes, probably due to widespread natural infections during childhood Costa Verdera et al., 2020. Moreover, patients who have previously received rAAV therapy often develop high titer of NAb that can persist for years George et al., 2020, significantly restricting the feasibility of second treatment with the same or different rAAV serotypes due to cross-reactivity. Various strategies are being explored to overcome these challenges, including rAAV capsid engineering to alter epitopes that are recognized by pre-existing antibodies (Tse et al., 2017), transient immunosuppressive (IS) agent to temporarily reduce antibody levels during the critical period of vector administration (Biswas et al., 2020; Karman et al., 2012), and plasmapheresis to physically remove antibodies from the circulation (Monteilhet et al., 2011). Since the above strategies are harmful to the human body, continued research into understanding and mitigating the effects of NAb will be essential for the future of gene therapy and its application in clinical settings.

In accordance with Chinese pharmacopeias, numerous traditional Chinese medicine (TCM) herbs exhibit immunomodulatory properties with high safety profiles and minimal side effects (Ren et al., 2017), and have become a focal point of current research (Yuan et al., 2022). *Artemisia annua L*., commonly known as sweet wormwood or Qinghao in TCM, has attracted considerable scientific interest due to its bioactive compounds, particularly artemisinin and its derivative dihydroartemisinin (DHA). These compounds, initially recognized for their potent antimalarial properties (Dai et al., 2021), have recently been identified for their potential in modulating the immune responses, suggesting broader therapeutic applications in conditions characterized by excessive immune activation, such as rheumatoid arthritis and multiple sclerosis Gao et al., 2024. DHA, in particular, has demonstrated inhibitory effects on various immune cells, including macrophages (Li et al., 2006), dendritic cells, and T cells (Chen et al., 2020). However, the effects of DHA on B cells have been relatively underexplored. Recent studies have shown that DHA can reduce the number of splenic and circulatory B cells in mice infected with protozoan parasites (Zhang et al., 2020) and ameliorate collagen-induced arthritis through the suppression of B cell activation (Hu et al., 2022). Additionally, Diao et al. demonstrated that co-delivery of DHA and siRNA targeting high mobility group box 1 (HMGB1) into lupus nephritis mice resulted in suppressed proliferation and activation of B cells through the TLR4 signaling pathway (Diao et al., 2022). These findings suggest that DHA could be a promising therapeutic agent for conditions involving excessive antibody production, prompting further investigation into its potential for reducing anti-AAV NAb.

In this paper, we demonstrate that DHA, a derivative extracted from TCM, significantly reduces anti-rAAVDJ NAb in mouse serum. Notably, DHA offers the advantages of low toxicity and minimal side effects, while maintaining the biodistribution and level of rAAV-mediated transgene expression. These results highlight DHA as a promising new IS agent for mitigating NAb in gene therapy.

## Materials and methods

### Cell culture

The human embryonic kidney cell line HEK293 was purchased from the American Type Culture Collection (ATCC, Manassas, United States). HEK293 cells were maintained in DMEM (Wisent, Montreal, Canada), supplemented with 10% FBS (Wisent, Montreal, Canada), and 1% penicillin-streptomycin (Wisent, Montreal, Canada). HEK293 cells were cultured in a Cell Incubator at 37°C, 5% CO_2_.

### rAAV vector production

rAAV vectors were obtained by using a PEI-mediated triple-plasmid transfection method (gene of interest, rAAVDJ capsid protein plasmid, and pAAV-helper) as previously described (Zheng et al., 2021). Briefly, HEK293 cells were harvested 72 h (hrs) post-transfection, and the cell pellet was resuspended in 5 mL of RB TMS Buffer (50 mM Tris-HCl, 150 mM NaCl, pH 8.0). After repeating a cycle of freezing in a dry-ice-ethanol bath for 10 min (mins) and thawing at 37°C for 10 min for three times, the lysate was centrifuged at 3,000 rpm for 10 min at 4°C, and the supernatant was collected for Benzonase digestion (EMD Millipore, Darmstadt, Germany). The supernatant was then layered on an iodixanol gradient (Sigma-Aldrich Inc., St. Louis, United States) in a 13 mL Quick-Seal centrifuge tube (Beckman Coulter, Brea, United States) and ultra-centrifuged at 75,000 rpm for 1 h. The 40% iodixanol fraction was collected, and the vectors were further purified by ion exchange chromatography using a HiTrap Q column (GE Healthcare, Piscataway, United States). After washing with phosphate-buffered saline, the vectors were concentrated using centrifugal spin concentrators with a 150 K molecular-weight cutoff and resuspended in 500 μL PBS (Wisent, Montreal, Canada). All vectors were subjected to quantitative real-time polymerase chain reaction (qPCR) to test the titer.

### Animal studies

The six-week-old male wild-type (WT) C57BL/6 mice were purchased from SLAC Laboratory (Shanghai, China). All mice were housed in an environment of 12 h light/dark cycle with controlled humidity (60%–80%) and temperature (22°C ± 1°C). All procedures were performed according to the guidelines for animal care specified by the Animal Care Services at Fudan University (Shanghai, China). The study and included experimental procedures were approved by the institutional animal care and use committee of Fudan University (approval no.2023JS009). All institutional and national guidelines for the care and use of laboratory animals were followed.

The rAAVDJ-CBAp-*gluc* vectors were diluted in 200 μL of PBS and injected intravenously via the tail vein as previously described (Xie et al., 2023), DHA (Targetmol, Shanghai, China), Paeoniflorin (Pae, Yuanye Bio-Technology Co. Ltd., Shanghai, China), Shaoyao Gancao Decoction (SGD, Wanshicheng Pharmaceutical Co. Ltd., Shanghai, China), Ganzaoning Decoction (GZND, Wanshicheng Pharmaceutical Co. Ltd., Shanghai, China) and Prednisolone (Pred, MedChemExpress, New Jersey, United States) in 1% hydroxy propyl-methyl cellulose (HPMC) and 0.5% Tween 80 (Sangon Biotech Co. Ltd., Shanghai, China) with PBS were intragastrically administrated.

### Anti-AAV NAb assay

The NAb assay was performed as previously described (Jungmann et al., 2017). Briefly, on day 1, 96-well plates were seeded with 5 × 10^4^ HEK293 cells per well for 24 h. The rAAVDJ-CMVp-*fluc* vectors were diluted in serum-free DMEM and incubated with serial dilutions of the serum samples for 30 min at 37°C. Subsequently, the serum-vector mixtures were added to the cells at a multiplicity of infection (MOI) of 1,000. After 48 h, cells were lysed by using a One-Lite Luciferase Assay Kit (Vazyme, Nanjing, China), and luciferase activity was measured on a luminometer (BioTek, Winooski, United States). Luciferase expression was measured as relative light units (RLUs) per second. The NAb titer was reported as the highest serum dilution that inhibited rAAV transduction by ≥ 50% compared to the 100% transduction control.

### Flow cytometry analysis

Lymphocytes were isolated from blood and spleens by using Red Blood Cell (RBC) Lysis Buffer (Biolegend, San Diego, United States). CD20^+^ B cells were stained with anti-CD20 antibody (Biolegend, San Diego, United States), CD19^+^GL7^+^ GC B cells were stained with anti-CD19 antibody (Biolegend, San Diego, United States) and anti-GL7 antigen antibody (Biolegend, San Diego, United States), CD19^+^CD138^+^ plasma cells were stained with anti-CD19 antibody (Biolegend, San Diego, United States) and anti-CD138 antibody (ABclonal, Wuhan, China). Samples were acquired on a Gallios flow cytometer (Beckman Coulter, Indianapolis, United States). The frequency of CD20^+^ B cells, CD19^+^GL7^+^ GC B cells and CD19^+^CD138^+^ plasma cells were analyzed by FlowJo version X.0.7 software (Tree Star, Ashland, United States).

### rAAV vector genome copy number quantification.

Total DNA was extracted from mouse tissues by an Animal Genomic DNA Extraction Kit (Tsingke, Beijing, China). Absolute quantification of vector genome copy number per 100 ng of DNA was determined by qPCR using a SYBR Green Kit (Tsingke, Beijing, China) with ITR-primers (Supplementary Table S1). 1, 0.1, and 0.01 ng of plasmid rAAV-*CMVp-gfp* were used to make the standard curve. And vector genome copy number was calculated by standard curve.

### qPCR analysis

Total RNA was isolated from spleens by using the MiniBEST Universal RNA Extraction Kit (Takara, Shiga, Japan) quantified by a Nanodrop. Equal amounts of RNA samples were used for reverse transcription by PrimeScrip RT Master Mix (Takara, Shiga, Japan). qPCR was performed by using SYBR Green (Tsingke, Beijing, China) with primers listed in Supplementary Table S1.

### rAAV vector transduction and DHA interference *in vitro*

HEK293 cells were seeded in 96-well plates at a final density of 5 × 10^4^ cells/well in complete DMEM. rAAVDJ-*fluc* vectors were transduced at an MOI of 1,000 vg/cell in free DMEM. After 2 h of transduction, the cells were added to complete DMEM. After 24 h of transduction, DHA was added, and the treatment for 24 h. The luciferase activity was measured using a luminometer (BioTek, Winooski, United States).

### Statistical analyses

Data are represented as the group mean ± standard deviations. Tests were performed on SPSS 20.0 software, using different statistical methods depending on the dataset to be analyzed and the experimental setup. The statistical methods applied are specified in the figure legends. P < 0.05 was considered significant.

## Results

### Effective decrease of pre-existing anti-rAAVDJ NAb in mice using DHA

**FIGURE 1:**
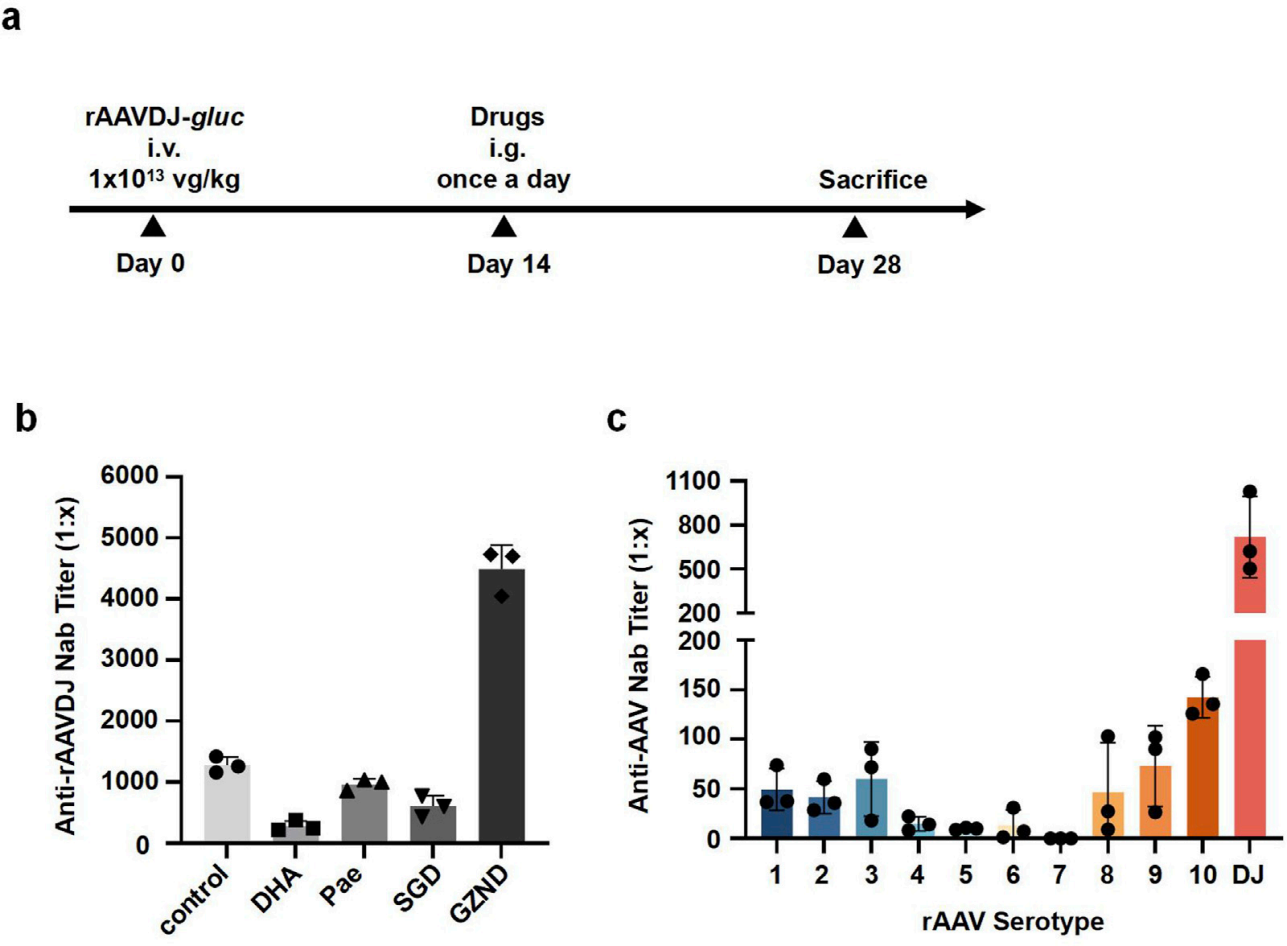
NAb titer against rAAVDJ in different drug-treated groups of C57BL/6 mice. **(a)** Experimental protocol. C57BL/6 mice were received 1 × 10^13^ vg/kg of rAAVDJ -*gluc*, then given PBS, 125 mg/kg DHA, 200 mg/kg Pae, 8.37 g/kg SGD, or 7.74 g/kg GZND in a volume of 200 μL per day on day 7 for 14 days. On day 21, mice were sacrificed, serum samples from drug-treated groups and control group were assayed. **(b)** Anti-rAAVDJ NAb titer analysis for control, DHA, Pae, SGD and GZND group. **(c)** The cross-reactivity between Anti-rAAVDJ NAb and serotypes 1-rh10 of rAAV. Serum from rAAVDJ challenged mice were co-incubated with serotypes 1-rh10 of rAAV vectors, respectively. The NAb titer of serotypes 1-rh10 of rAAV was determined.

To demonstrate proof-of-concept for the effect of TCM on pre-existing NAb against rAAV, we immunized six-week-old WT C57BL/6 mice with an intravenous injection of rAAVDJ-*gluc* vectors at a dose of 1 × 10^13^ vg/kg. Starting 14 days post-injection (PI), the rAAVDJ-immunized mice received daily intragastric administration of either 125 mg/kg DHA, 200 mg/kg Pae, 8.37 g/kg SGD, or 7.74 g/kg GZND in a volume of 200 μL for 14 consecutive days. On day 28 PI, the mice were sacrificed, and NAb titer in serum was assayed (Figure 1a). Appreciable titer of anti-rAAVDJ NAb was observed in the control mice as well as in the Pae-, SGD- and GZND-treated groups, with titer of 1,282 ± 131, 963 ± 93, 611 ± 240, and 4,493 ± 387, respectively. In contrast, the DHA-treated mice exhibited significantly lower levels of anti-rAAVDJ NAb, with titer of 284 ± 86 (Figure 1b).

To further test cross-reactivity with other rAAV serotypes, the serum from rAAVDJ-challenged mice was incubated with rAAV serotypes 1–9, rh10, and DJ at 37°C for 30 min. The results indicated that the NAb titer for serotype DJ was approximately 1:800, followed by serotypes 1, 2, 3, 8, 9, and rh10, which had NAb titer around 1:100. Serotypes 4, 5, 6, and seven showed the least NAb, with titer less than 1:20 (Figure 1c). This data suggests that there is minimal cross-reactivity between NAb against rAAVDJ and other rAAV serotypes.

### 125 mg/kg DHA administration is optimal for safety in mice

**FIGURE 2:**
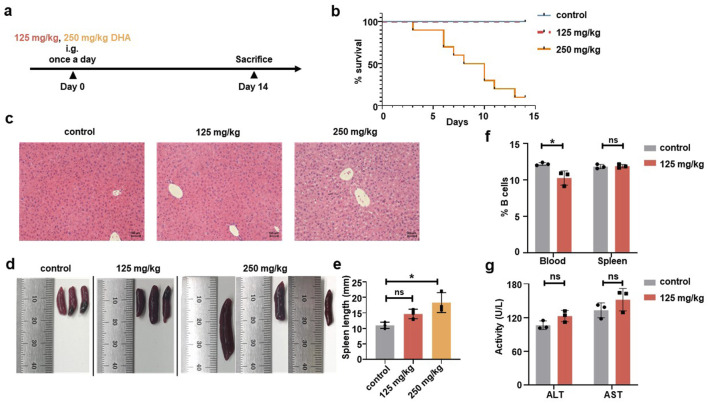
Different doses of DHA were intragastrically administered to C57BL/6 mice. **(a)** Experimental protocol. C57BL/6 mice were given PBS, 250 mg/kg or 125 mg/kg DHA in a volume of 200 μL for 14 days by once a day (n = 10). Mice were sacrificed for following detection. **(b)** A survival curve of different DHA doses. The number of mice alive or died were recorded for the group of 250 mg/kg and 125 mg/kg DHA as well as the control group. **(c)** Representative liver sections were stained by H&E and observed under a microscope (original magnification ×20). **(d)** Representative images of spleens for the control group, 125 mg/kg DHA group and 250 mg/kg DHA group. **(e)** Spleen length was measured to analysis Splenomegaly. **(f)** The change of CD20^+^ B cells frequency from blood and spleen for both control group and the 125 mg/kg DHA group by Flow cytometry. **(g)** The serum levels of ALT and AST were detected both control group and the 125 mg/kg DHA group. Statistical analyses were performed by analysis of variance (ANOVA) in e, and by unpaired t-test in f, g (*p < 0.05, ns no significance).

To determine the optimal dose, six-week-old WT C57BL/6 mice (n = 10) received daily intragastric administration of either PBS, 125 mg/kg DHA, or 250 mg/kg DHA in a volume of 200 μL for 14 consecutive days (Figure 2a). Previous research has demonstrated that 125 mg/kg DHA can produce significant therapeutic effects in mice with systemic lupus erythematosus (Li et al., 2006). Survival analysis revealed that the mice in the 125 mg/kg DHA group maintained a 100% survival rate, comparable to the control group, while the 250 mg/kg DHA group exhibited a drastic reduction, with only 20% survival by the end of the administration period (Figure 2b; Supplementary Table S2). Successfully, we obtained liver and spleen samples from the last three mice in each group for evaluating the pharmacological toxicity effects of DHA. Hematoxylin and eosin (H&E) staining showed that the liver tissue from the 125 mg/kg DHA group and the control group was normal, with no signs of cell enlargement or nuclear abnormalities (Figure 2c). In contrast, the liver tissue from the 250 mg/kg DHA group displayed disordered cellular distribution, indicative of liver damage. Both DHA-treated groups exhibited splenomegaly compared to the control, with a dose-dependent increase in spleen size (Figures 2d,e). Due to unexpected mortality in the 250 mg/kg DHA group, lymphocyte analysis was conducted only for the other groups. Flow cytometry analysis indicated that 125 mg/kg DHA treatment resulted in a slight decrease in the frequency of CD20^+^ B cells in the blood, but not in the spleen (Figure 2f), suggesting that in the absence of specific antigens, DHA administration does not significantly impact B cell frequency. Notably, serum levels of alanine aminotransferase (ALT) and aspartate aminotransferase (AST) in the 125 mg/kg DHA group did not differ significantly from that in the control group, indicating minimal liver toxicity at this dose (Figure 2g).

### DHA administration after short-term rAAVDJ delivery decreased anti-rAAVDJ NAb in mice

**FIGURE 3:**
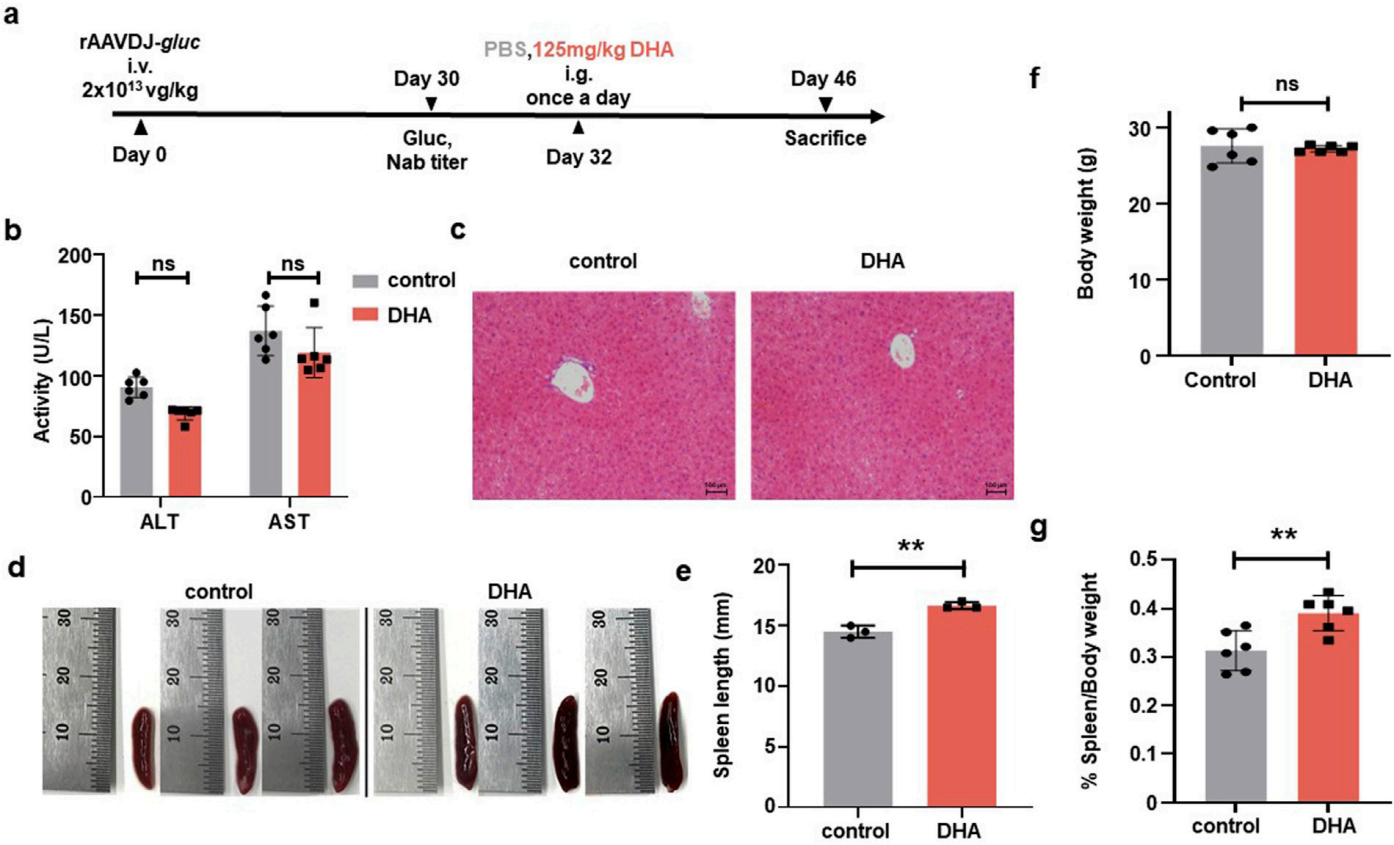
Toxicological damage of the DHA administration after rAAVDJ having delivered 30 days. **(a)** Experimental protocol. C57BL/6 mice were received 2 × 10^13^ vg/kg of rAAVDJ-*gluc* on day 0 by intravenous injection. Gluc expression and NAb titer was determined on day 30. C57BL/6 mice were given intragastric administration of PBS or 125 mg/kg DHA in a volume of 200 μL on day 32 for 14 days by once a day, respectively. On day 46, mice were sacrificed to following detection (n = 6). **(b)** The serum levels of ALT and AST were detected at the end of administration for the control group and the DHA group. **(c)** Representative liver sections were stained by H&E and observed under a microscope (original magnification ×20). **(d)** Representative images of spleens control group and the DHA group. **(e)** Spleen length was measured. **(f)** Body weights were measured before and after administration for control group and the DHA group. **(g)** Spleen weight and body weight were measured and the ratio of spleen weight to body weight were analyzed. Statistical analyses were performed by unpaired t-test in b, e, f, g (**p < 0.01, ***p < 0.001, ns no significance).

To investigate the impact of DHA on humoral immunity in the context of short-term rAAV vector delivery, six-week-old WT C57BL/6 mice were injected via the tail vein with rAAVDJ-*gluc* vectors at a dose of 2 × 10^13^ vg/kg (n = 6). On day 30 PI, we initially measured the anti-rAAVDJ NAb titer in mice and observed significant variation among individuals, which may be attributed to differences in individual immunity. Based on the initial NAb titer, mice were randomly assigned to the control group and the DHA group. Starting on day 32 PI, mice received daily intragastric administration of either PBS or 125 mg/kg DHA in a volume of 200 μL for 14 consecutive days. The mice were sacrificed on day 46 PI (Figure 3a). In comparison with the control group, the DHA-treated group showed no significant changes in serum levels of ALT and AST (Figure 3b). Liver and spleen samples from three mice in each group were collected for H&E staining and further splenic analysis. The H&E staining of liver tissue did not reveal any abnormal changes in liver cords or hepatocytes (Figure 3c). Notably, the spleen length in the DHA-treated group was slightly increased compared to the control group (Figures 3d,e). On the day of sacrifice, both body and spleen weights were recorded, showing that DHA-treated mice had comparable growth to the control mice (Figures 3f,g). In summary, our findings suggest that 125 mg/kg DHA has minimal toxicological effects on the growth of rAAV-treated mice.

**FIGURE 4:**
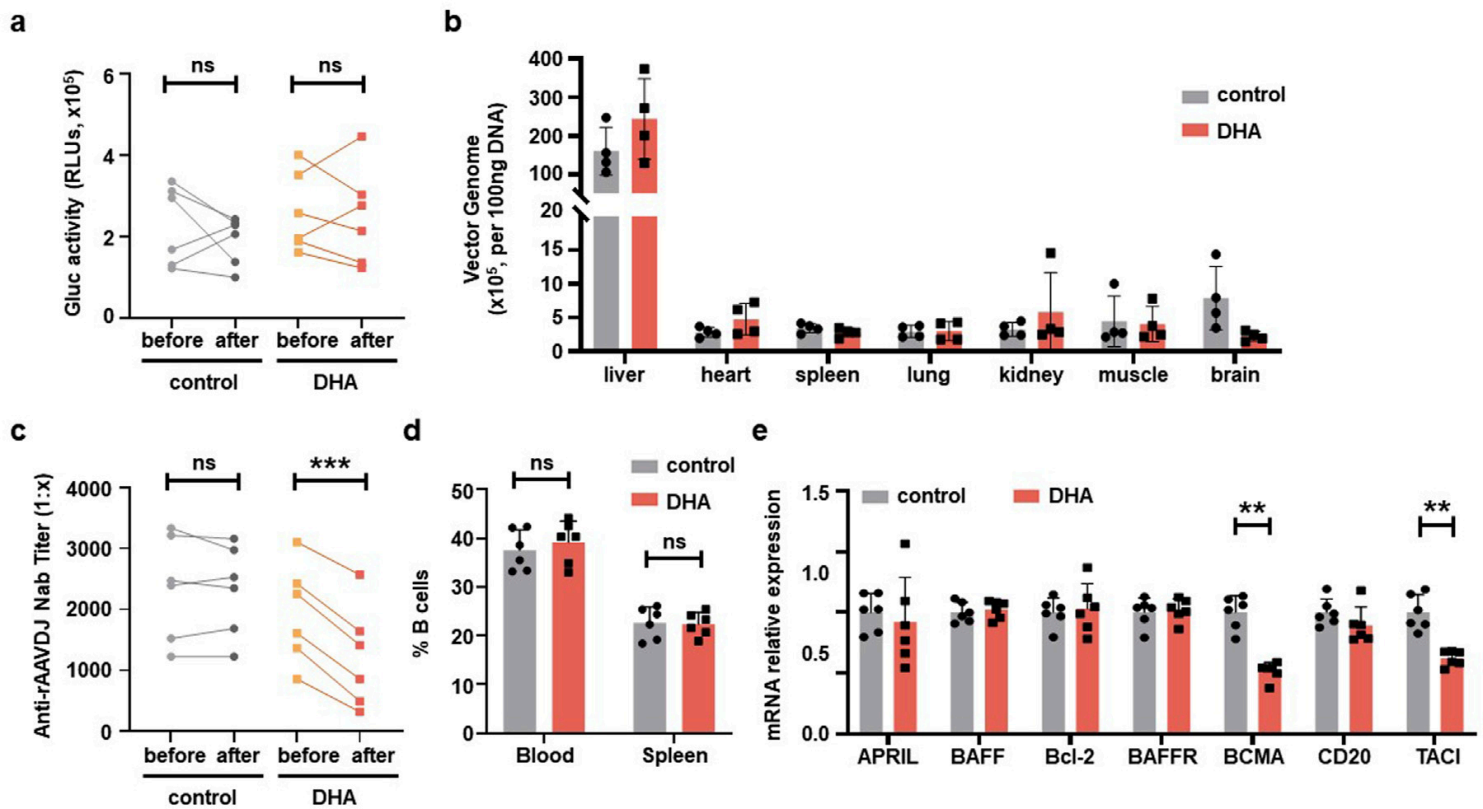
DHA administration after rAAVDJ having delivered 30 days. **(a)** Gluc transgene expression analysis before and after DHA administration for both control group and the DHA group. **(b)** Biodistribution of vector genomes were detected at the end of administration for control group and the DHA group. **(c)** Anti-rAAVDJ NAb titer analysis before and after administration for both control group and the DHA group. **(d)** The change of CD20^+^ B cells frequency from blood and spleen at the end of administration for both control group and the DHA group. **(e)** mRNA expression analysis of genes related to B cell in spleen, including April, BAFF, Bcl-2, BAFFR, BCMA, CD20 and TACI. QPCR was used to determine the amount of mRNA. Statistical analyses were performed by paired t-test in a, c, and by unpaired t-test in b, d, e (**p < 0.01, ***p < 0.001, ns no significance).

To evaluate the transduction efficiency of rAAVDJ vectors in mice, we first measured the levels of Gluc in mouse serum. There was no significant difference between the 125 mg/kg DHA group and the control group before and after DHA administration (Figure 4a). Regarding biodistribution, we collected major tissues from four mice in each group, the copy number of vector genomic DNA of the DHA group showed no changes across organs compared with the control group. The liver retained the majority of the rAAVDJ vector genomic DNA. Therefore, we concluded that DHA administration does not impact rAAV *in vivo* transduction (Figure 4b). Most importantly, after the DHA intervention, we observed that NAb titer in the control group remained unchanged, while the DHA group exhibited a dramatic decrease, regardless of the initial NAb titer (Figure 4c). For instance, a high pre-treatment titer of 1:2,251 decreased to 1:1,414 following DHA administration, while a lower initial titer of 1:861.2 was further reduced to 1:323.8. It was reported that the reduction in NAb is often associated with a decrease in B cell numbers (Holodick et al., 2017). However, our flow cytometry results showed no significant difference in the frequency of B cells in the blood and spleen between the DHA and the control group (Figure 4d). These findings prompt us to investigate the relative mRNA expression of genes related to B lymphocyte proliferation in the spleen, including a proliferation-inducing ligand (April), B cell activating factor (BAFF), B cell lymphoma-2 (Bcl-2), B cell activating factor receptor (BAFFR), BCMA, cluster of differentiation 20 (CD20), and transmembrane activator and CAML interactor (TACI). The results (Figure 4e) indicated no significant changes in the expression of April, BAFF, Bcl-2, and BAFFR in the DHA-treated group compared to the control group, suggesting no alteration in mature B cell numbers. However, BCMA and TACI expression was decreased in the DHA group, which may account for the subsequent lower NAb levels. The specific mechanisms underlying these observations warrant further investigation.

### DHA administration after long-term rAAVDJ delivery decreased anti-rAAVDJ NAb in mice

**FIGURE 5:**
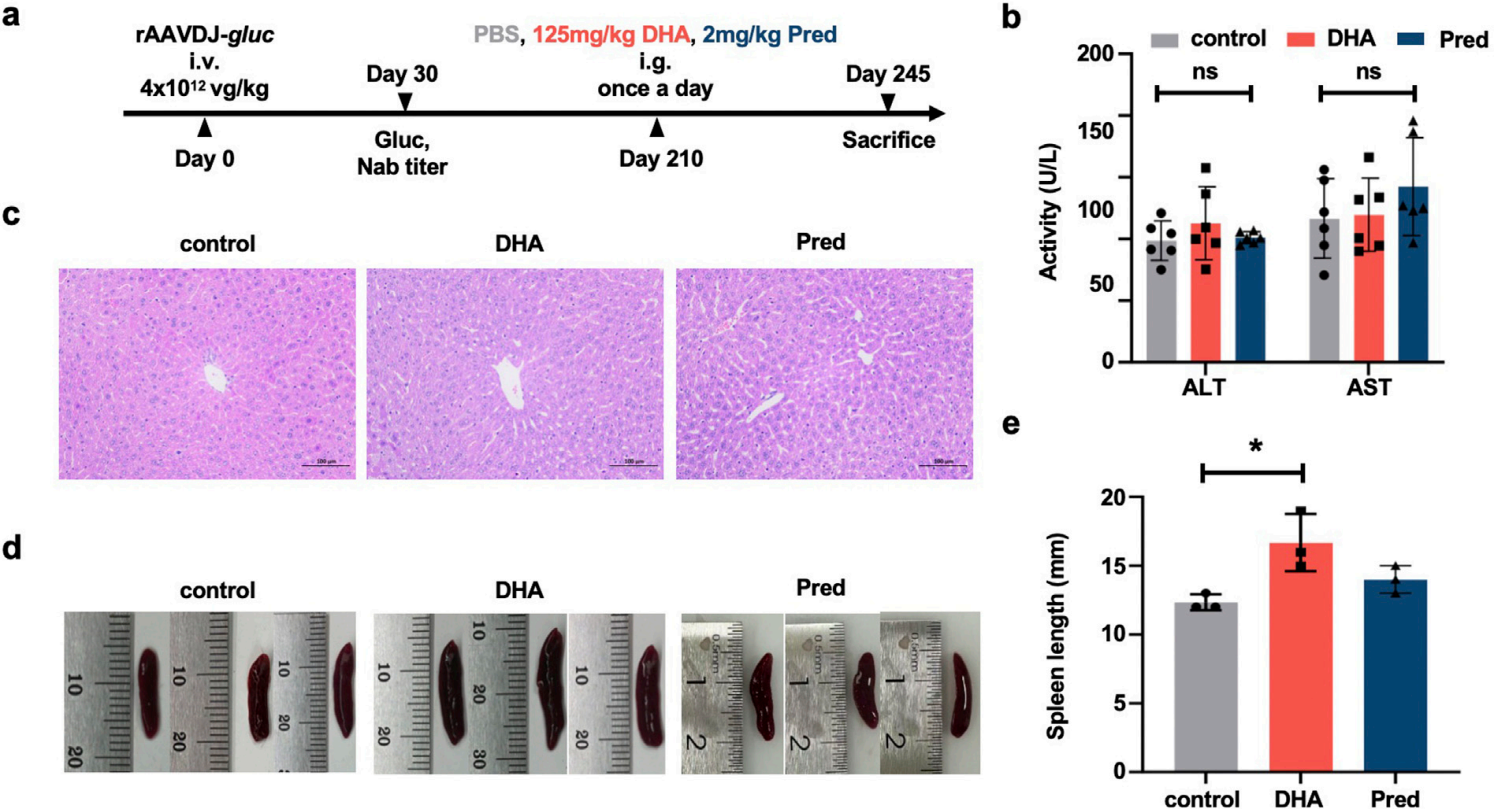
Toxicological damage of the DHA administration after rAAVDJ having delivered 210 days. **(a)** Experimental protocol. C57BL/6 mice were received 4 × 10^12^ vg/kg of rAAVDJ-*gluc* on day 0 by intravenous injection. Gluc expression and NAb titer was determined on day 30. C57BL/6 mice were given intragastric administration of PBS, 125 mg/kg DHA or 2 mg/kg prednisolone in a volume of 200 μL on day 210 for 35 days by once a day, respectively. On day 245, mice were sacrificed to following detection (n = 6). **(b)** The serum levels of ALT and AST were detected at the end of administration. **(c)** Representative liver sections were stained by H&E and observed under a microscope (original magnification ×20). **(d)** Representative images of spleens. **(e)** Spleen length was measured. Statistical analyses were performed by ANOVA in b, e (*p < 0.05, ns no significance).

To investigate the impact of DHA on humoral immunity in the context of long-term rAAV vector delivery, and to compare its efficacy with the commonly used IS agent prednisolone (Pred) (Velazquez et al., 2017), six-week-old WT C57BL/6 mice were injected via the tail vein with rAAVDJ-*gluc* vectors at a dose of 4 × 10^12^ vg/kg (n = 6). Beginning on day 210 PI, mice received daily intragastric administration of either PBS, 125 mg/kg DHA, or 2 mg/kg Pred in a volume of 200 μL for 35 consecutive days. The mice were sacrificed on day 245 PI (Figure 5a). The results showed no significant difference in ALT and AST levels among the control, DHA, and Pred groups (Figure 5b). Liver and spleen samples from three mice in each group were collected for analysis. H&E staining revealed that the liver cells exhibited intact cellular structures, tightly arranged cells, and centrally located nuclei across all groups (Figure 5c). Consistent with the findings from the short-term delivery study (Figures 3d,e), the DHA-treated group displayed slight spleen enlargement compared to the control group after 35 days of continuous DHA administration (Figures 5d,e).

**FIGURE 6:**
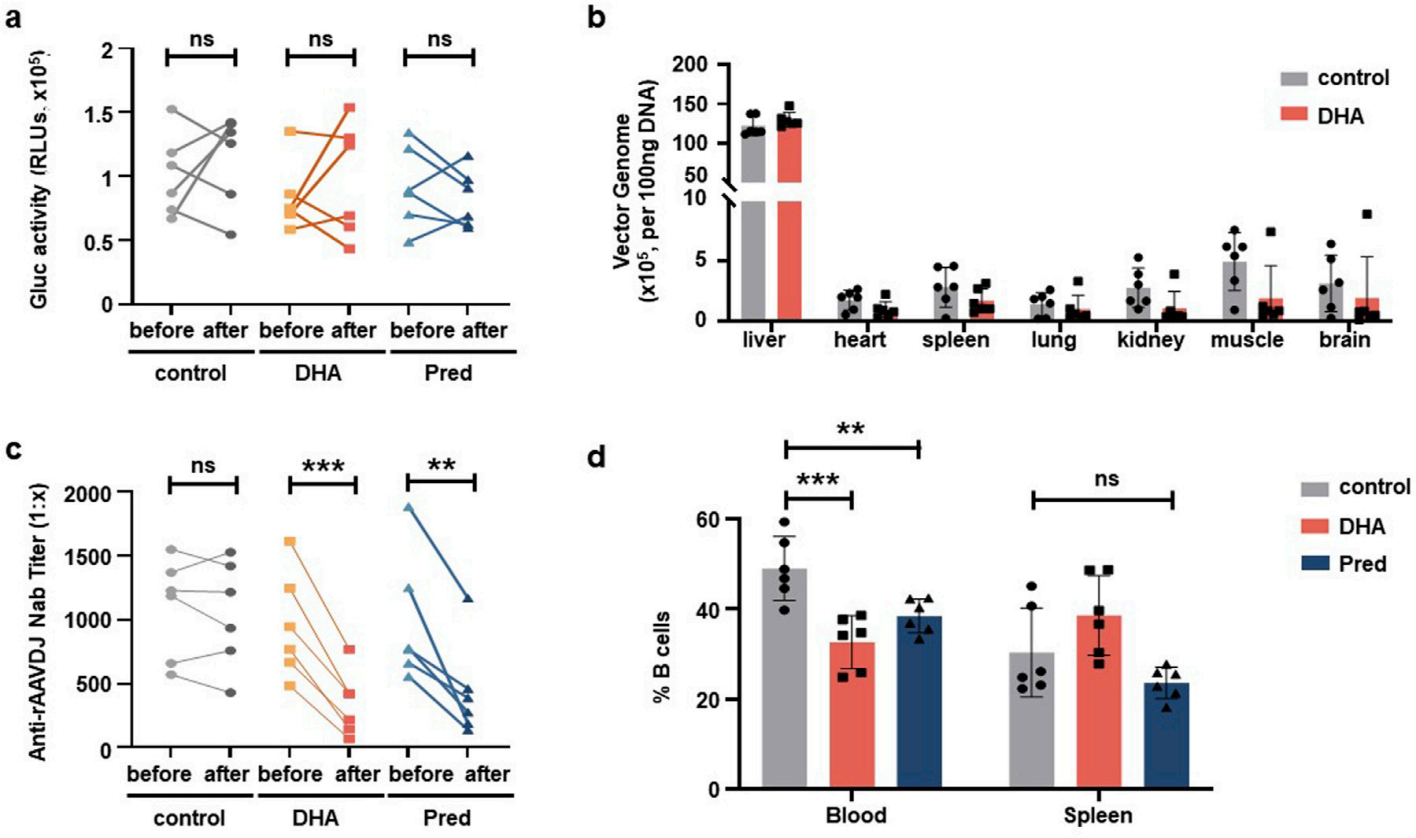
DHA administration after rAAVDJ having delivered 210 days. **(a)** Gluc transgene expression analysis before and after administration. **(b)** Biodistribution of vector genomes were detected at the end of administration for control group and the DHA group. **(c)** Anti-rAAVDJ NAb titer analysis before and after administration. **(d)** The change of CD20^+^ B cells frequency from blood and spleen at the end of administration. Statistical analyses were performed by paired t-test in a, c, by unpaired t-test in b, and by ANOVA in d (**p < 0.01, ***p < 0.001, ns no significance).

**FIGURE 7:**
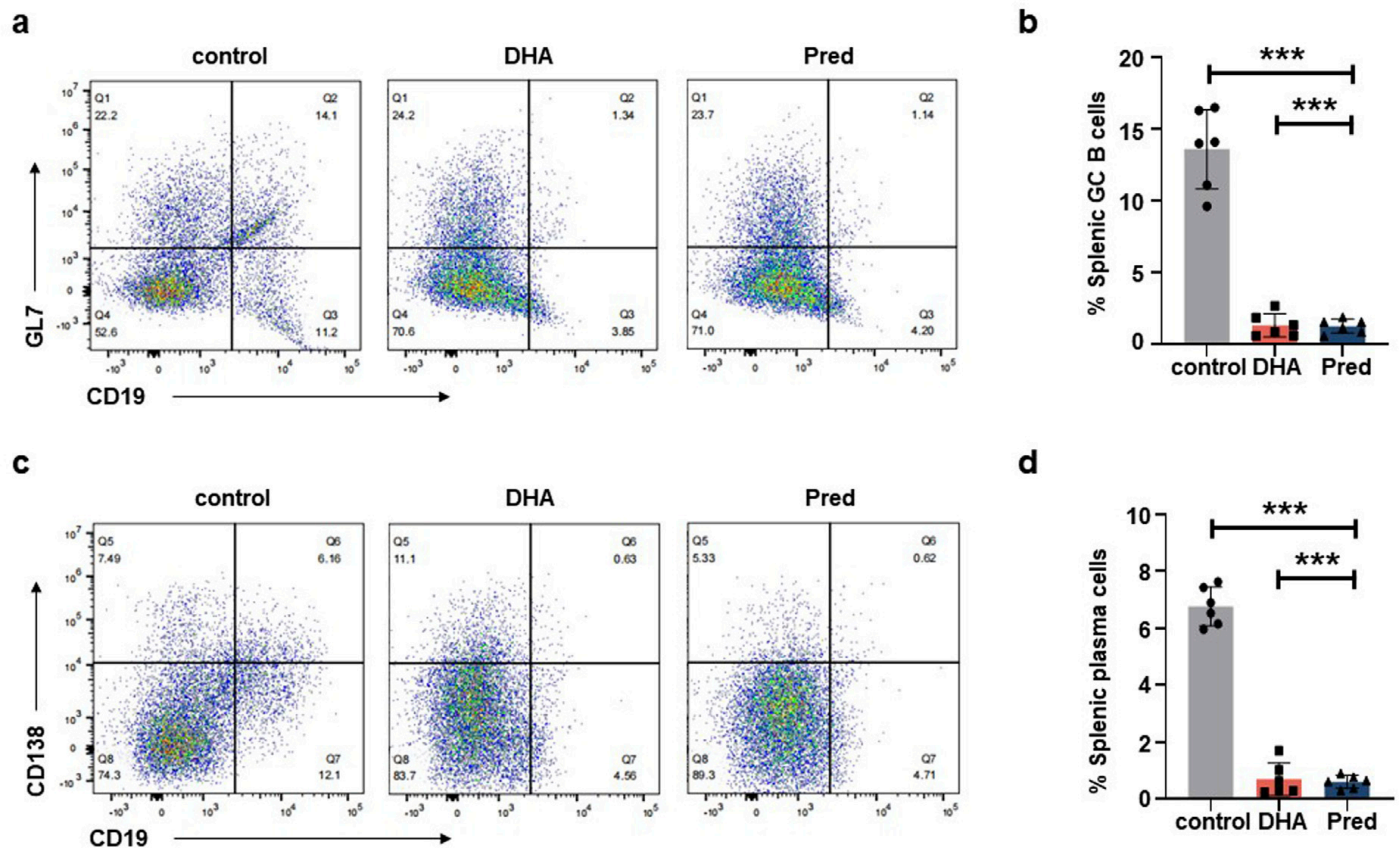
DHA administration reduce splenic GC B cells and plasma cells after rAAVDJ having delivered 210 days. **(a)** Representative flow cytometry plots of splenic CD19^+^GL7^+^ GC B cells at the end of administration. **(b)** The change of splenic CD19^+^GL7^+^ GC B cells frequency at the end of administration. **(c)** Representative flow cytometry plots of splenic CD19^+^CD138^+^ plasma cells at the end of administration. **(d)** The change of splenic CD19^+^CD138^+^ plasma cells frequency at the end of administration. Statistical analyses were performed by ANOVA in b, d (***p < 0.001, ns no significance).

Next, we assessed serum Gluc levels, vector biodistribution, anti-rAAVDJ NAb titer, and CD20^+^ B cell frequency. The levels of serum Gluc remained unchanged among the control, Pred and DHA groups (Figure 6a) and the biodistribution of rAAVDJ vectors were similar between the control and DHA groups (Figure 6b), consistent with the findings from the short-term DHA intervention in rAAV vector delivery. Notably, NAb titer in the DHA group decreased significantly, from the highest initial NAb titer 1:1,615 to 1:767 or from the lowest initial NAb titer 1:482 to 1:66, a trend comparable to that observed in the Pred group (Figure 6c). It is noteworthy that the frequency of CD20^+^ B lymphocytes in the blood of the 125 mg/kg DHA group as well as Pred group markedly decreased compared to the control group (Figure 6d), which correlates with the observed reduction in NAb levels. To further investigate the underlying mechanism, we examined the effects of long-term rAAVDJ delivery on splenic germinal center (GC) B cells and plasma cells. Flow cytometry analysis revealed a marked reduction in the proportion of CD19^+^GL7^+^ GC B cells in the spleens of mice treated with DHA or Pred compared to the control group (Figures 7a,b). Similarly, the frequency of CD19^+^CD138^+^ plasma cells was significantly lower in both the DHA and Pred groups (Figures 7c,d).

### DHA has no inhibitory effect on rAAV transduction *in vitro*

**FIGURE 8:**
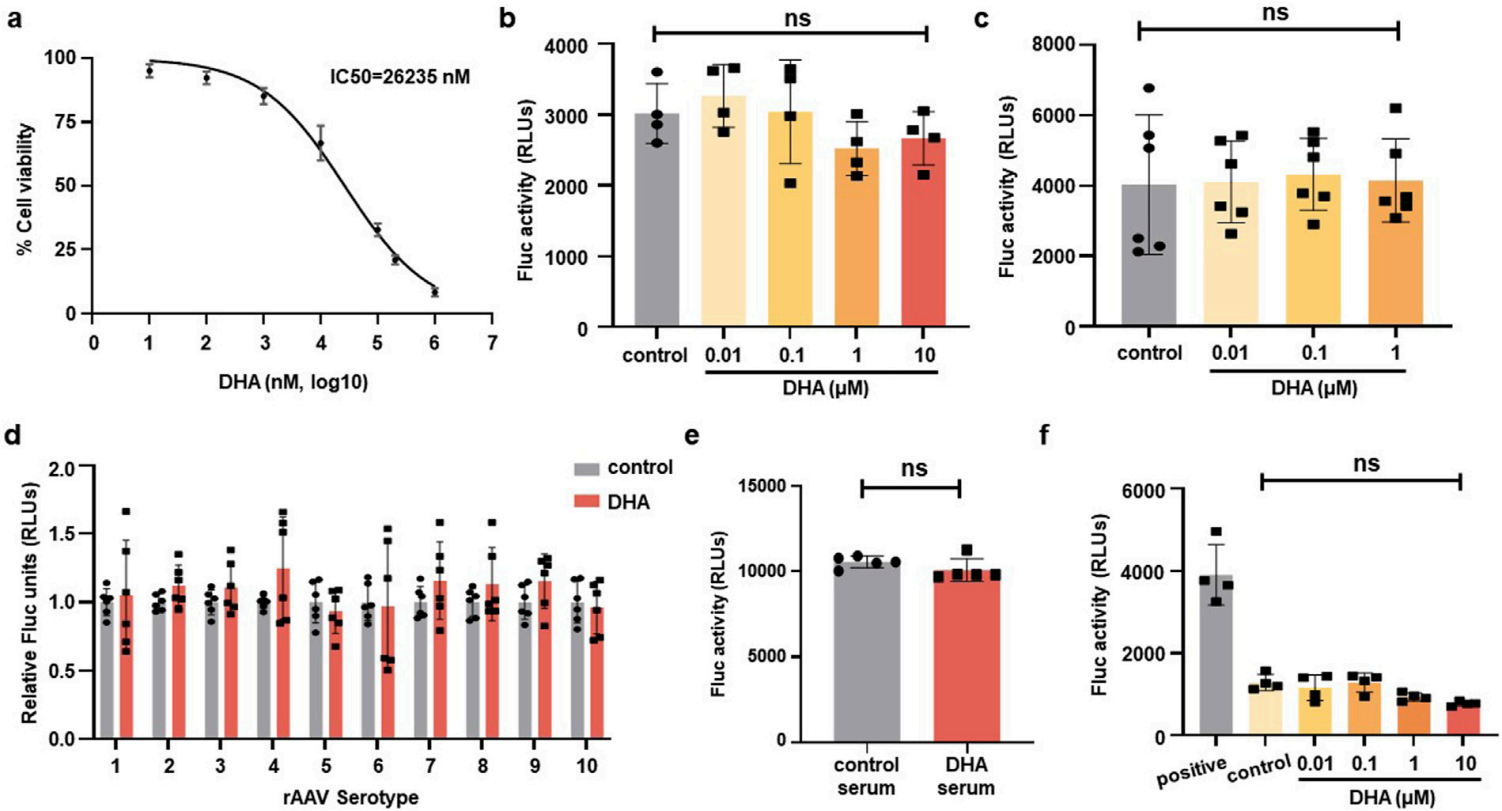
DHA has no inhibiting effect on rAAV infection on HEK293 cells. **(a)** HEK293 cells were treated with indicated concentrations of DHA for 72 h, followed by CCK-8 assays to evaluate cell viability. IC50 values (26,235 nM) were calculated. **(b)** After rAAVDJ infected HEK293 cells at MOI = 1,000 vg/cell for 24 h, DHA with indicated doses were added for 24 h. Fluc expression were detected. **(c)** DHA with indicated doses were co-infected with rAAVDJ at MOI = 1,000 vg/cell for 72 h on HEK293 cells. Fluc expression were detected. **(d)** The serotypes 1-rh10 of rAAV were used to infect HEK293 cells at MOI = 1,000 vg/cell. At the same time, 1 μM DHA was added to co-infect for 72 h. Fluc expression were detected. **(e)** The influence of serum from DHA treated mice on rAAV infection. Serum from the DHA group and the control group were diluted to 1:100 and co-incubated with rAAVDJ for HEK293 cells infection. **(f)** DHA with indicated doses were co-infected with rAAVDJ at MOI = 1,000 vg/cell and serum containing anti-rAAVDJ Nab for 72 h on HEK293 cells. Fluc expression were detected. Statistical analyses were performed by ANOVA in b, c, and by unpaired t-test in d, e, f (*p < 0.05, ***p < 0.001, ns no significance).

To further investigate the effect of DHA on rAAV transduction, *in vitro* studies were performed using HEK293 cells. After 72 h intervention, cell viability was assessed using the CCK-8 reagent, which revealed that the IC50 of DHA on HEK293 cells is roughly 26 μM (Figure 8a). Next, HEK293 cells were transduced with rAAVDJ-*fluc* vectors for 24 h, followed by treatment of increasing doses of DHA from 0.01 to 10 μM. Alternatively, HEK293 cells were transduced with rAAVDJ-*fluc* vectors for 72 h, co-treated with increasing doses of DHA from 0.01 to 1 μM. The results showed that varying concentrations of DHA did not significantly affect the rAAV transduction efficiency compared to the control group (Figures 8b,c). Next, we examined the impact of 1 μM DHA on other rAAV serotype vectors, including rAAV1-9 and rh10. After 72 h of DHA treatment, no significant changes in Fluc expression was observed across all serotypes (Figure 8d), suggesting that DHA does not interfere with Fluc expression *in vitro*.

We also investigated whether DHA metabolites in blood would affect the transgene expression of rAAV vectors. Serum from five mice treated with 125 mg/kg DHA for continuous 14 days was collected, diluted 100-fold, and incubated with rAAVDJ-*fluc* vectors at 37°C for 30 min. Serum from mock-treated mice served as a control. HEK293 cells were then transduced with rAAVDJ vectors at an MOI of 2,000 vg/cell for 72 h to assess Fluc expression. The results showed that serum from DHA-treated mice did not influence rAAV-mediated transgene expression (Figure 8e).

To rule out the roles of DHA on preventing capsid-antibody interaction, we incubated increasing doses of DHA from 0.01 to 10 μM with equal amounts of NAb-containing serum. This mixture was then used to treat HEK293 cells with an equal amount of rAAVDJ-*fluc*. We included a positive control group with only rAAVDJ-*fluc* and a control group with serum and rAAVDJ-*fluc* but without DHA. The results showed that the fluc activity in the positive control group was significantly higher than in all other groups, confirming the inhibitory effect of NAb in the serum on rAAV transduction. There was no significant difference between the control group without DHA and the DHA groups at different doses, ruling out the possibility that DHA alter the capsid-antibody interaction (Figure 8f).

## Discussion

To our knowledge, this is the first report demonstrating the novel function of DHA in reducing pre-existing anti-AAV NAb. We hypothesize that this inhibitory effect is not specific to AAV, but rather indicative of a broader IS action that affects B cell proliferation. Notably, DHA administration in the context of long-term rAAV delivery led to a reduction in peripheral B cells, along with decreased frequencies of splenic GC B cells and plasma cells. These results were not observed in the short-term rAAV delivery, which may be explained by the fact that AAV infection can induce an increase in B cell levels in the short term (Xiang et al., 2022). Previous report has shown that AAV vectors rapidly activate innate immune responses, leading to the release of pro-inflammatory cytokines such as IL-1 and IL-6, which promote B cell proliferation, activation, and differentiation (Calcedo et al., 2018). Consistently, a recent study demonstrated a measurable expansion of B cell populations within 7 days of AAV injection (Ilyinskii et al., 2023). Furthermore, Wang et al. reported that AAV capsids accumulate in splenic germinal centers and persist for at least 35 days, providing sustained antigenic stimulation that supports early B cell activation (Wang et al., 2011). This increase may potentially counteract the B cell-lowering effect of DHA during the early phase of treatment. Although no significant changes in B cell counts were observed, DHA treatment after short-term rAAV exposure resulted in a reduction of B cell-related gene expression, specifically BCMA and TACI. Previous studies have scarcely explored the relationship between DHA and B cell function or antibody production. Zhao et al. suggested that DHA may regulate the balance between Th17 and Treg cell generation by modulating the mTOR signaling pathway, a mechanism that could indirectly influence B cell development (Zhao et al., 2012). Zhang et al. proposed that DHA may control Treg function and inhibit GC formation through activation of the SOD3-JNK-AP-1 signaling axis (Zhang et al., 2022). Moreover, Diao et al. reported that co-delivery of DHA and siRNA targeting HMGB1 inhibited B cell activation and differentiation (Diao et al., 2022), although the individual effects of DHA and siRNA were not specifically delineated. The proposed mechanisms differ across studies, further investigation is warranted to clarify how DHA, or its active metabolites, influences B cell function and antibody production.

Our findings revealed that DHA may induce splenomegaly in mice, accompanied by altered expression levels of specific genes in spleen tissue. The causes of splenomegaly are varied, including infectious factors such as bacterial or viral infections (Pozo et al., 2009) and non-infectious factors, predominantly liver cirrhosis and splenic congestion (Schwabl et al., 2022). It is hypothesized that high-dose (250 mg/kg) DHA administration might be linked to non-infectious splenomegaly, likely associated with liver damage. Histological analysis via H&E staining revealed disordered distribution of liver cells in the 250 mg/kg DHA-treated mice. Previous studies have shown an increase in the number and proportion of splenic neutrophils and lymphocytic infiltration in response to DHA treatment, which may contribute to spleen enlargement in low-dose (125 mg/kg) DHA-treated mice (Zhang et al., 2022). Splenomegaly is generally associated with an increased number of B cells. However, in our study, DHA treatment led to splenomegaly accompanied by a notable reduction in B cell levels. Interestingly, a recent study reported that B cell depletion can occur in the context of splenomegaly, potentially due to lymphocyte retention within the spleen or disruptions in homing pathways (Viallard et al., 2024). DHA may trigger a similar mechanism, although further research is required to clarify the underlying molecular processes. Moreover, studies have reported that mice with TACI gene defects exhibit splenomegaly (von Bülow et al., 2001), TACI deficiency is also associated with humoral immunodeficiency, particularly low IgM serum concentrations (Salzer et al., 2005). Further investigations are needed to clarify how DHA induces splenomegaly. The ultimate objective is to refine DHA or employ combination therapies to mitigate its side effects.

Mutations in rAAV capsid epitopes can significantly impact the tropism and transduction efficiency of rAAV vectors, making it challenging to be an easy feat to evade NAb (Li et al., 2022; Weber, 2021). To address this, researchers have explored various strategies to reduce NAb, including transient IS agents, plasmapheresis, and antibody-cleaving techniques Weber, 2021. While these approaches have shown promise in animal studies, translating them to clinical practice poses significant challenges due to their complexity and associated risks. Many of these strategies have been used clinically for other applications. For instance, corticosteroids are the most commonly used transient IS agents in AAV studies (Li et al., 2022), but their use can lead to severe side effects such as osteoporosis, adrenal suppression, and Cushing’s syndrome. Plasmapheresis can result in the non-specific removal of all circulating IgG, leading to hypogammaglobulinemia and an increased risk of infection (Ahmed and Kaplan, 2020), necessitating hospitalization for patients to manage potential complications. In our current investigation (Figure 1B), DHA was preliminarily identified as the most effective candidate for reducing NAbs. Although the initial screening was conducted with a relatively small sample size, it served as a proof-of-concept study and yielded results consistent with those observed in subsequent experiments. Future investigations will focus on expanding the screening library and increasing sample size to systematically identify novel TCM-derived candidates capable of reducing NAb. DHA has a well-established history as an effective antimalarial drug, noted for its minimal adverse effects, excellent safety profile, and stability, which has significantly improved patient outcomes. Our findings support the safety of DHA at therapeutic doses and demonstrate its efficacy in reducing anti-AAV NAb in both short-term and long-term rAAV administration in mice. Given these promising results, further evaluation of DHA in non-human primates is justified. Such research could expand the applicability of rAAV gene therapy to a wider patient population and potentially facilitate the re-administration of rAAV-based treatments.

## Conclusion

These studies highlight DHA as an effective IS agent with little toxicity for reducing NAb against rAAV vectors, offering proof-of-concept data for the use of TCM and TCM-derived drugs in addressing major challenges in gene therapy.

## Data Availability

The original contributions presented in the study are included in the article/Supplementary Material, further inquiries can be directed to the corresponding authors.
