# Supplementary material for: Dihydroartemisinin decreases pre-existing neutralizing antibodies against adeno-associated virus in challenged mice
Source: Front Pharmacol. 2025 Aug 1;16:1587135. doi: 10.3389/fphar.2025.1587135 (PMC12355050; doi:10.3389/fphar.2025.1587135)
Supplement: Supplementary file 1 [file DataSheet1.pdf]

Supplementary Table S1

Gene Name	Primer Sequence (5'→3')
APRIL	Forward: CTTTCGGTTGCTCTTTGGTTG<br>Reward: CGACAGCACAAGTCACAGC
BAFF	Forward: ACACTGCCCAACAATTCCTG<br>Reward: TCGTCTCCGTTGCGTGAAATC
BAFFR	Forward: TCTGGTGAGAAACTGCGTGTC<br>Reward: GTCAGCGCCAGTATCAGTCC
Bcl-2	Forward: ATGCCTTTGTGGAACTATATGGC<br>Reward: GGTATGCACCCAGAGTGATGC
BCMA	Forward: GGCGCAACAGTGTTTCCACA<br>Reward: CTCGGTGTCGGCCTTGTCCA
CD20	Forward: AACCTGCTCCAAAAGTGAACC<br>Reward: CCCAGGGTAATATGGAAGAGGC
TACI	Forward: ATGGCATTCTGCCCCAAAGAT<br>Reward: ATGGTCGTAGTACCTGCCTTG
beta-actin	Forward: CGAGGCCCAGAGCAAGAG<br>Reward: CCACACGCAGCTCATTGTA
ITR	Forward: GGAACCCCTAGTGATGGAGTT<br>Reward: CGGCCTCAGTGAGCGA
CMVp	Forward: CATTGACGTCAATAATGACG<br>Reward: TGTACTGGGCATAATGCCAG

Supplementary Table S2

Days	Number of surviving mice		
	control	DHA 125mg/kg	DHA 250mg/kg
1	10	10	10
2	10	10	10
3	10	10	9
4	10	10	9
5	10	10	9
6	10	10	7
7	10	10	6
8	10	10	5

9	10	10	5
10	10	10	3
11	10	10	2
12	10	10	2
13	10	10	1
